# The association of variants in *PNPLA3* and *GRP78* and the risk of developing hepatocellular carcinoma in an Italian population

**DOI:** 10.18632/oncotarget.13558

**Published:** 2016-11-24

**Authors:** Daniele Balasus, Michael Way, Caterina Fusilli, Tommaso Mazza, Marsha Y. Morgan, Melchiorre Cervello, Lydia Giannitrapani, Maurizio Soresi, Rosalia Agliastro, Manlio Vinciguerra, Giuseppe Montalto

**Affiliations:** ^1^ Biomedical Department of Internal Medicine and Medical Specialties, University of Palermo, Palermo, Italy; ^2^ Institute for Liver & Digestive Health, Division of Medicine, Royal Free Campus, University College London, London, UK; ^3^ IRCCS Casa Sollievo della Sofferenza, Bioinformatics Unit, San Giovanni Rotondo (FG), Italy; ^4^ Institute of Biomedicine and Molecular Immunology, National Research Council (C.N.R.), Palermo, Italy; ^5^ Immunohematology and Transfusion Medicine Unit, “Civico” Reference Regional Hospital, Palermo, Italy; ^6^ Center for Translational Medicine (CTM), International Clinical Research Center (ICRC), St. Anne's University Hospital, Brno, Czech Republic

**Keywords:** hepatocellular carcinoma, hepatitis C virus, single nucleotide polymorphisms, risk factors, genetic variants

## Abstract

Hepatocellular carcinoma (HCC) has one of the worst prognoses amongst all malignancies. It commonly arises in patients with established liver disease and the diagnosis often occurs at an advanced stage. Genetic variations, such as single nucleotide polymorphisms (SNPs), may alter disease risk and thus may have use as predictive markers of disease outcome. The aims of this study were (i) to assess the association of two SNPs, rs430397 in *GRP78* and rs738409 in *PNPLA3* with the risk of developing HCC in a Sicilian association cohort and, (ii) to use a machine learning technique to establish a predictive combinatorial phenotypic model for HCC including rs430397 and rs738409 genotypes and clinical and laboratory attributes. The controls comprised of 304 healthy subjects while the cases comprised of 170 HCC patients the majority of whom had hepatitis C (HCV)–related cirrhosis. Significant associations were identified between the risk of developing HCC and both rs430397 (p=0.0095) and rs738409 (p=0.0063). The association between rs738409 and HCC was significantly stronger in the HCV positive cases. In the best prediction model, represented graphically by a decision tree with an acceptable misclassification rate of 17.0%, the A/A and G/A genotypes of the rs430397 variant were fixed and combined with the three rs738409 genotypes; the attributes were age, sex and alcohol. These results demonstrate significant associations between both rs430397 and rs738409 and HCC development in a Sicilian cohort. The combinatorial predictive model developed to include these genetic variants may, if validated in independent cohorts, allow for earlier diagnosis of HCC.

## INTRODUCTION

Hepatocellular carcinoma (HCC) is the most common of the primary liver cancers. It is the fifth most common malignancy worldwide and the third most frequent cause of cancer-related deaths [1–3]. The epidemiology of HCC is complex reflecting, to a large extent, differences in levels of exposure to known predisposing factors such as the presence of cirrhosis, chronic infection with hepatitis B virus (HBV) and hepatitis C virus (HCV) and environmental toxin exposure. Prevalence rates are 16 to 32 times higher in economically less- developed countries such as sub-Saharan Africa South-east Asia and China [4]. In Eastern Asia the major risk factor for HCC is infection with hepatitis B [5, 6], whereas in Northern and Western Europe excess alcohol consumption and infection with HCV are the main antecedents [3]. In developed countries HCC develops most frequently in patients with established cirrhosis whereas elsewhere HCC may arise in patients with chronic HBV and HCV infection at a pre-cirrhotic stage [7, 8]. There are also data to support a role for tobacco smoking, diabetes and obesity as risk factors or risk co-factors for the development of HCC [9]. Finally there are important gender differences in HCC prevalence; men are at two- to five-fold increased risk of developing this malignancy than women, depending on geographic area, which most likely reflect their increased exposures to known risk factors [1].

As the epidemiology of HCC has become better understood, so has the fact that only 5 to 20% of the populations potentially at risk actually develop HCC. Interest has, therefore, turned to the identification of the factors responsible for the differences in individual susceptibility; in particular the role of genetic variation. Two of the most notable genetic findings to date have been the association between the Single Nucleotide Polymorphisms (SNP), rs430397 and rs738409, with HCC risk. The rs430397 variant in glucose regulated protein 78 (GRP78 also known as HSPA5) has been associated with HCC and cirrhosis risk in Chinese populations with HBV infection [10, 11]. There is considerable functional and genetic evidence implicating the GRP78 protein in mechanisms of HCC carcinogenesis [12, 13] and indeed in malignancies at other sites [14, 15]. The rs738409 variant in patatin-like phospholipase domain-containing 3 (*PNPLA3*) has been identified as conferring a significant risk for developing cirrhosis in relation to non-alcohol-related fatty liver disease [16] and alcohol-related liver disease [17]. The rs738409 variant not only contributes to liver injury, but also to the risk of development of subsequent HCC [18, 19]. The nature of the association between the rs738409 variant in HCV-related HCC is less well characterized than in HCC of other aetiologies possibly because there is significant heterogeneity in the study populations in which this variant has been studied [20].

Variants in *GRP78* and *PNPLA3* have been associated with HCC risk in populations of diverse ancestry and with HCC of diverse aetiology. It is not known, however what proportion of the variance in HCC risk can be attributed to genetic factors in Southern European populations in whom the main risk factor for the development of HCC is chronic HCV infection and the incidence is rising [9]. Further, it is widely recognized that HCC risk prediction in chronic liver disease will have clinical utility in guiding evidence-based decisions about patient management [21] but it is unclear as to whether genetic information could improve the predictive models. Machine learning techniques have the potential to improve the accuracy of predictive models using genetic data [22]. A number of machine learning techniques have been developed, but all make use of computer algorithms that improve predictive accuracy with experience. Decision tree algorithms in particular are a well-established technique, which are useful for exploratory analysis and model visualisation.

The aims of the present study were therefore to assess, whether (i) two genetic variants in PNPLA3 and GRP78 are associated with the risk of developing HCC in a Sicilian population, and (ii) whether inclusion of genotypic information would contribute to a predictive model designed to stratify patients by HCC risk.

## RESULTS

### Cohort characteristics

The cases were significantly older than the healthy controls (mean ± 1SD age 70.2± 8.0 vs. 56.6 ± 8.1 years; p = 2.2 × 10^-16^) and comprised of proportionately fewer men (70.4% vs. 58.2%, p = 0.007) (Table 1). All but one of the cases had established cirrhosis; in the majority chronic HCV infection was the aetiological agent (Table 1). A small proportion of patients (n=12; 7%), had marked steatosis in addition to cirrhosis. Up to 40% of the cases had experienced a major complication of chronic liver disease; and the majority had features of hepatocellular dysfunction with poor synthetic and excretory function (Table 2). Features of cirrhosis were evident on imaging in all included cases; ultrasonography was the modality most frequently employed (Table 2). The diagnosis of HCC was made in the majority on the basis of multimodal imaging; less than a quarter of patients had an elevated serum alpha-fetoprotein level (Table 3).

**Table 1:** Characteristics of the healthy controls and HCC cases included in the genetic association study

Variable	Controls (n=304)	HCC cases (n=170)
*Age at diagnosis (yr)*	56.6 ± 8.1	70.2 ± 8.0
*Gender*		
Men	214 (70.4)	99 (58.2)
Women	90 (29.6)	71 (41.8)
*Aetiology of Liver Disease*		
HCV		144 (84.7)
Alcohol		10 (5.9)
Cryptogenic		10 (5.9)
HBV		9 (5.3)
Dysmetabolic syndrome		1 (0.6)

Data are mean ± 1SD or number (%)

Abbreviations: HCV- hepatitis C virus; HBV- hepatitis B virus.

**Table 2:** Historical, clinical, laboratory, imaging and histological information used to diagnosis cirrhosis in the HCC cases

Variables used to diagnose cirrhosis	Cases (n=170)
*History and clinical findings*	Affected/abnormal n (%)
Variceal hemorrhage	66 (38.8)
Ascites	44 (25.9)
Hepatic encephalopathy	10 (5.9)
*Laboratory investigations*	
Thrombocytopaenia*	137 (80.6)
Prolonged prothrombin time*	53 (31.2)
Hypoalbuminaemia*	13 (7.6)
Hyperbilirubinaemia*	11 (6.5)
*Investigations including imaging*	
Ultrasound	163 (95.9)
CT-Scan	8 (4.7)
Endoscopy	5 (2.9)
*Histology*	20 (11.8)

* Laboratory cut-off values: platelet count: < 150 × 10^9^/L, Prothrombin time > 40 sec, plasma albumin < 35 g/L, serum bilirubin > 1.2 mg/dl.

**Table 3:** Laboratory, imaging and histological information used to diagnose HCC

Variables used to diagnose cirrhosis	Cases (n=170)
*Laboratory Investigations*	Affected/abnormal n (%)
Elevated serum alpha-fetoprotein*	37 (21.8)
*Imaging*	
CT-Scan	146 (85.9)
Ultrasound	27 (15.9)
MRI-Scan	27 (15.9)
*Histology*	20 (11.8)

* Laboratory cut-off values: serum alpha-fetoprotein > 400 ng/ml.

### Genotyping quality

The genotyping success rate was greater than 95% for both variants, with genotype distributions showing no evidence for deviation from Hardy-Weinberg equilibrium in controls (p >0.05). The minor and major allele designations of both rs430397 and rs738409 were generally similar in this Sicilian ancestry cohort to those in reference Europeans ancestry groups [23]; however, the minor allele (G) of rs738409 was noticeably more frequent in Sicilian controls than in European reference populations.

### GRP78 and PNPLA3 polymorphisms and HCC

Both rs430397 in *GRP78* and rs738409 in *PNPLA3* were significantly associated with HCC risk in this Sicilian ancestry cohort (Table 4). The association between rs430397 in *GRP78* and HCC risk is best described by a dominant model (AA+AG vs. GG), (p= 0.0095, OR= 1.81) whereas the association between rs738409 in *PNPLA3* is best described by a recessive model (GG vs. CG +CC), (p= 6.28×10^-3^, OR= 2.56) (Table 5).

**Table 4:** Allelic associations between the SNPs rs430397 and rs738409 in sicilian cases with HCC and healthy controls

Gene (SNP)	Group	n	Minor Allele	Genotype Counts			MAF	Cases vs Controls	
								Significance p	OR (95% CI)
				AA	AG	GG			
*GRP78*(rs430397)	Cases	170	A	1	46	123	0.141	0.016	1.65(1.09-2.50)
	Controls	304			2	51	251	0.090	
				GG	CG	CC			
*PNPLA3*(rs738409)	Cases	170	G	35	64	71	0.39	4.22×10^-3^	1.50 (1.14-1.98)
	Controls	304		28	128	148	0.30		

Abbreviations: SNP – Single nucleotide polymorphism; n – number; MAF – Minor allele frequency; OR: Odds ratio; CI: Confidence Interval.

**Table 5:** Associations between rs430397 and rs738409 genotypes in sicilian cases with HCC and healthy controls using different genetic models

Gene (SNP)	Model	Entire cohort (n=474)			Entire cohort excluding non HCV-related cases (n=448)		
		*P*	OR	95% CI	*P*	OR	95% CI
	Allelic	0.014	1.71	1.11-2.63	0.0348	1.64	1.04-2.58
*GRP78*(rs430397)	Dominant	0.0095	1.81	1.16-2.83	0.0190	1.76	1.10-2.82
	Recessive	0.93	0.89	0.08-9.93	-	-	-
	Allelic	0.0065	1.45	1.11-1.90	0.00363	1.52	1.15-2.02
*PNPLA3*(rs738409)	Dominant	0.15	1.32	0.91-1.93	0.127	1.37	0.92-2.04
	Recessive	0.0063	2.56	1.49-4.38	2.41×10^-3^	2.82	1.62-4.90

Abbreviations: SNP – Single nucleotide polymorphism; OR: Odds ratio; CI: Confidence Interval.

When the analyses were confined to cases with HCC related to chronic HCV infection there was no evidence of association between rs430397 in *GRP78* and HCC risk (Table 5). Because the frequency of the rs430397: AA genotype in this stratified cohort was very low, an association test under a recessive model of inheritance (AA vs. AG+GG) could not be performed using logistic regression. In contrast, the magnitude of the association between rs738409 in *PNPLA3* and HCC risk increased when confined to the HCV positive HCC cases (p=2.41×10^-3^, OR=2.82) (Table 5). There was no evidence for an epistatic interaction between the two variants and HCC risk (P_asymptotic_ = 0.8, OR_interaction_ = 1.08). None of the demographic, clinical, laboratory or diagnostic variables was associated with either the *GRP78* or *PNPLA3* genotypes (Table 6).

**Table 6:** Association between rs430397 and rs738409 and demographic, clinical and laboratory variables in the HCC cases

Characteristics	rs430397			rs738409				
	A/A + G/A (n=47)	G/G (n=123)	*p*	C/C (n=71)	C/G (n=64)	G/G (n=35)	*p* (C/C vs C/G)	*p* (C/C vs G/G)
Age (years):								
<71	21 (44.7%)	59 (48%)		35 (49.3%)	31 (48.4%)	14 (40%)	0.942	0.4867
>=71	26 (55.3%)	64 (52%)	0.832	36 (50.7%)	33 (51.6%)	21 (60%)		
Gender:								
M	23 (48.9%)	76 (61.8%)		44 (62%)	38 (59.4%)	17 (48.6%)	0.895	0.270
F	24 (51.1%)	47 (38.2%)	0.178	27 (38%)	26 (40.6%)	18 (51.4%)		
HCV:								
Yes	39 (83%)	105 (85.4%)		59 (83.1%)	53 (82.8%)	32 (91.4%)	0.853	0.389
No	8 (17%)	18 (14.6%)	0.882	12 (16.9%)	11 (17.2%)	3 (8.6%)		
HBV:								
Yes	3 (6.4%)	6 (4.9%)		5 (7%)	3 (4.7%)	1 (2.9%)	0.831	0.667
No	44 (93.6%)	117 (95.1%)	0.993	66 (93%)	61 (95.3%)	34 (97.1%)		
Alcohol:								
Yes	3 (6.4%)	7 (5.7%)		4 (5.6%)	6 (9.4%)	0 (0%)	0.617	0.374
No	44 (27.5%)	116 (94.3%)	0.847	67 (94.4%)	58 (90.6%)	35 (100%)		
Cirrhosis:								
Yes	47 (100%)	122 (99.2%)		71 (100%)	64 (100%)	34 (97.1%)	0.606	0.717
No	0 (0%)	1 (0.8%)	0.616	0 (0%)	0 (0%)	1 (2.9%)		
Cryptogenic:								
Yes	4 (8.5%)	6 (4.9%)		4 (5.6%)	4 (6.2%)	2 (5.7%)	0.831	0.667
No	43 (91.5%)	117 (95.1%)	0.592	67 (94.4%)	60 (93.7%)	33 (94.3%)		
Dysmetabolic:								
Yes	0 (0%)	1 (0.8%)		0 (0%)	1 (1.6%)	0 (0%)	0.958	7.00×10^-3^
No	47 (100%)	122 (99.2%)	0.616	71 (100%)	63 (98.4%)	35(100%)		
Ascites:								
Yes	11 (23.4%)	33 (26.8%)		22 (31%)	12 (18.8%)	10 (28.6%)	0.958	0.976
No	36 (76.6%)	90 (73.2%)	0.795	49 (69%)	52 (81.2%)	25 (71.4%)		
Variceal hemorrhage:								
Yes	16 (34%)	50 (40.7%)		28 (39.4%)	19 (29.7%)	19 (54.3%)	0.314	0.215
No	31 (66%)	73 (59.3%)	0.539	43 (60.6%)	45 (70.3%)	16 (45.7%)		
Hepatic encephalopathy:								
Yes	1 (2.1%)	9 (7.3%)		6 (8.5%)	2 (3.1%)	2 (5.7%)	0.345	0.912
No	46 (97.9%)	114 (92.7%)	0.357	65 (91.5%)	62 (96.9%)	33 (94.3%)		
Thrombocytopaenia:								
Yes	42 (89.4%)	95 (77.2%)		55 (77.5%)	52 (81.5%)	30 (85.7%)	0.742	0.458
No	5 (10.6%)	28 (22.8%)	0.116	16 (22.5%)	12 (18.8%)	5 (14.3%)		
Prolonged prothrombin time								
Yes	16 (34%)	37 (30.1%)		23 (32.4%)	20 (31.2%)	10 (28.6%)	0.966	0.860
No	31 (66%)	86 (69.9%)	0.754	48 (67.6%)	44 (68.7%)	25 (71.4%)		
Hyperbilirubinaemia								
Yes	5 (10.6%)	6 (4.9%)		3 (4.2%)	5 (7.8%)	3 (8.6%)	0.606	0.643
No	42 (89.4%)	117 (95.1%)	0.309	68 (95.8%)	59 (92.2%)	32 (91.4%)		
Hypoalbuminaemia								
Yes	4 (8.4%)	9 (7.3%)		4 (5.6%)	7 (10.9%)	2 (5.7%)	0.418	0.667
No	43 (91.5%)	114 (92.7%)	0.952	67 (94.4%)	57 (89.1%)	33 (94.3%)		
Raised serum alphafetoprotein								
Yes	8 (17%)	29 (23.6%)	0.472	16 (22.5%)	16 (25%)	5 (14.3%)	0.894	0.458
No	39 (83%)	94 (76.4%)		55 (77.5%)	48 (75%)	30 (85.7%)		

### Modelling of phenotypes and PNPLA3/GRP78 polymorphisms in the HCC cases

**Figure 1:**
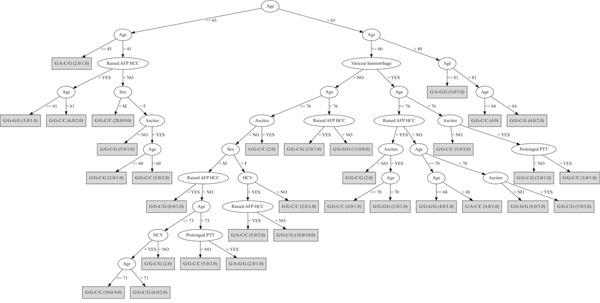
Decision tree based on the genotypes of both PNPLA3 and GRP78 SNPs In this analysis the included discriminating attributes were: age, sex, HCV status, ascites, variceal haemorrhage, prolonged prothrombin time (PTT), elevated serum alpha-fetoprotein (AFP). The first genotype refers to the rs430397 variant and the second, separated by “-”, refers to the rs738409 variant. The ratio of the genotypes accurately classified over those wrongly classified is provided for each genotype in brackets.

The first decision tree included different combinations of rs430397 and rs738409 genotypes, and the attributes of: age, sex, HCV status, ascites, variceal haemorrhage, prolonged prothrombin time and elevated serum alpha-fetoprotein (Figure 1). This decision tree had a misclassification rate (MCR) of 40%.

**Figure 2:**
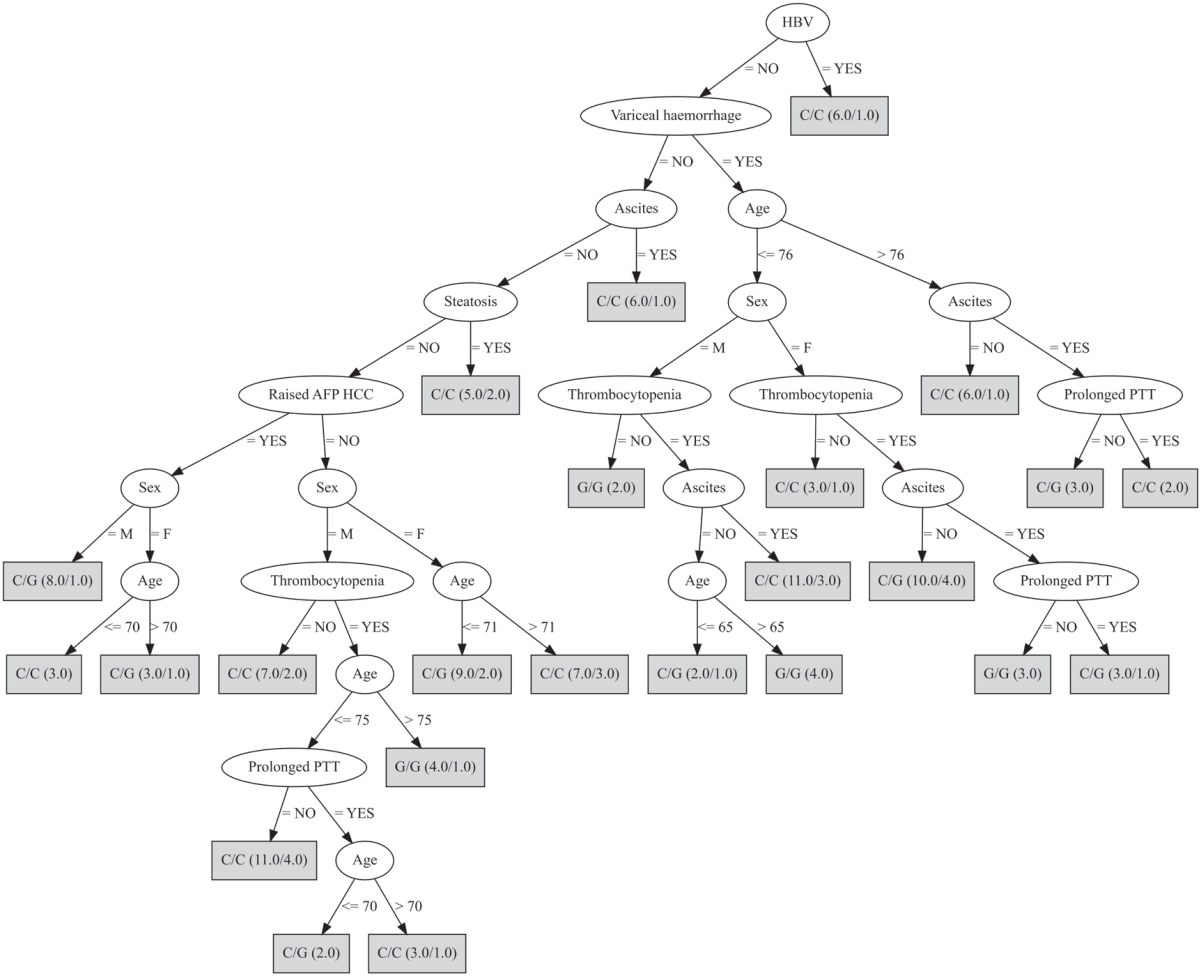
Decision tree developed fixing the rs430397 G/G genotype In this analysis, the most discriminating attributes were: age, sex, HBV, steatosis, ascites, variceal haemorrhage, thrombocytopenia, prolonged prothrombin time (PTT), elevated serum alpha-fetoprotein (AFP). The genotype in the box refers to rs738409. The ratio of the genotypes accurately classified over those wrongly classified is provided for each genotype in brackets.

The second decision tree included only three possible genotypic combinations: the rs430397 G/G was fixed and combined with the three rs738409 genotypes; the discriminating attributes were age, sex, HBV status, steatosis, ascites, variceal haemorrhage, thrombocytopenia, prolonged prothrombin time, elevated serum alpha-fetoprotein (Figure 2). The predictive power of this decision tree substantially improved resulting in a MCR of 24.4%.

**Figure 3:**
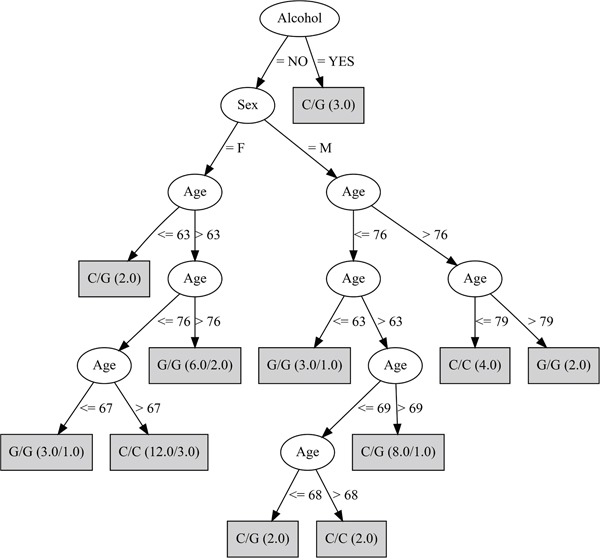
Decision tree developed fixing the rs430397 A/A and G/A genotypes This analysis selected the age, sex and alcohol variables. The genotype in the box refers to the rs738409 SNP. The ratio of the genotypes accurately classified over those wrongly classified is provided for each genotype in brackets.

The third decision tree, which was complimentary to the second, kept only A/A and G/A genotypes of the rs430397 variant and the discriminating attributes: age, sex and alcohol. This was the most discriminating of the decision trees with a MCR of 17.0% (Figure 3).

## DISCUSSION

The high variability of HCC incidence worldwide undoubtedly relates to differences in the distribution of environmental risk factors and most likely to variations in genetic susceptibilities between ethnic groups [24]. Outcomes in patients with HCC are critically dependent on early detection and diagnosis and hence the efficacy of public health strategies and screening programs [5]. The success of these programs, in turn, depends on the availability of validated, predictive markers with high sensitivity and specificity.

In the present study, two SNPs, rs430397 in *GRP78* and rs738409 in *PNPLA3*, were shown to be significantly associated with the risk of developing HCC in a Sicilian population. Further the possibility that these risk-associated genetic variants could be used to prediction the development of HCC on an individual patient basis was explored, using a machine learning technique, with promising results.

Rs430397 lies in the fifth intron of *GRP78*. It has been associated with HCC risk in Chinese with HBV infection [10, 11] but does not appear to have been studied in European populations previously. As a non-protein-coding SNP, it could influence gene/protein function by altering gene expression but there is no direct evidence, to date, that this variant has functional effects. However, it does not lie in a *GRP78* gene region associated with high transcriptional activity [25] nor in a CpG island associated with epigenetic mechanisms of gene silencing (T. Mazza: personal communication October 2016).

Physiologically the GRP78 protein acts as a molecular chaperone, which is activated by endoplasmic reticulum stress [26] and is involved in intracellular calcium ion homeostasis [27]. It has also been reported to sustain cell survival, to inhibit apoptosis and to promote the invasion and metastasis of HCC cells *in vitro* [28–31]. However, the precise role of GRP78 in the development of cancer is still not clear [32, 33].

Rs738409 lies in the third exon of *PNPLA3* and encodes a nonsynonymous alteration in the protein sequence (Ile148Met). This SNP came to prominence when identified as a risk factor for the progression of non-alcohol related fatty liver disease [16] and the development of alcohol-related cirrhosis [17]. Subsequently, the risk allele of rs738409 was shown to be associated with the development of HCC in patients of European descent with established cirrhosis [20]. Rs738409 has also been associated with the development of HCC in East Asian populations [34, 35]. The risk allele of rs738409 has also been studied in relation to HCV-related liver injury, although associations are less consistent [36, 37]. The results of the present study show that the rs738409:G allele is a significant risk for HCC in the Sicilian population. The fact that the strength of the association was increased in the subgroup with HCV-related cirrhosis with an effect size that exceeds that in many other studies is of interest but has to be weighed against the fact that these individuals comprised almost 85% of the study population.

Despite the substantial genetic evidence implicating rs738409 and *PNPLA3* with cirrhosis and HCC of different aetiologies, the function of the PNPLA3 protein remains uncertain as does the effect of the Ile148Met substitution. The 148Met risk allele appears to promote intracellular triglyceride retention [38] but the functional changes that lead to the development of significant liver damage and HCC remain to be established. Of interest the association between rs738409 and liver disease progression appears to be independent of the severity of liver fat accumulation [20, 39]. Further, the functional effects of this variant may directly or indirectly regulate the release of molecules involved in inflammation and fibrogenesis as intercellular adhesion molecule 1 and adiponectin [40–43].

The MAF of the rs738409 variant in the Sicilian control population utilized in the present study was higher than expected when compared with an ancestrally appropriate European reference population, e.g. the Toscani from Italy [23] (30% cf. 23%). This difference in allele frequency could reflect genetic isolation of the population of Sicily. However, a recent study has shown that Sicilians are genetically similar to mainland Italians from the adjacent regions [44]. Another potential explanation for this observation could be cryptic underlying population stratification, which may have arisen due to the use of blood-donor controls. Despite this, similar blood-donor controls are used in the Wellcome Trust Case-Control consortium cohort [45] with little evidence to suggest significant population stratification in their analyses.

There was no evidence, in the present study, of an epistatic interaction between the *GRP78* and *PNPLA3* variants in relation to HCC risk. In addition there were no significant associations between either variant and any clinical features or the results of any of the laboratory investigation. However, combinations of these various attributes together with the rs430397 and rs738409 genotypes were used to produce a model, graphically displayed as a decision tree, which could be useful for predicting subjects at risk for developing HCC, at least within this study population. The best prediction model used only age, sex and alcohol as the additional required variables and was represented by a decision tree with a MCR of 17.0%.

In conclusion: rs430397 in *GRP78* and rs738409 in *PNPLA3* are risk factors for the development of HCC in a Southern Italian population of cases with predominantly HCV-related cirrhosis. Use of a machine-learning approach allowed development of a prediction model incorporating phenotypic, clinical and genotypic variables. This computational approach needs to be further explored and the predictions independently validated. If confirmed, this approach could be used to identify individuals at risk at an early stage thereby facilitating monitoring and, when required, early intervention.

## PATIENTS AND METHODS

### Study populations

The 170 HCC cases were enrolled at the Department of Internal Medicine and Medical Specialties of the Policlinico Hospital, Palermo, Italy. All had been born in Sicily and continued to reside there. The aetiology of the liver disease was determined using historical, clinical, laboratory, imaging and histological information. The diagnosis of cirrhosis and HCC were made based on previously reported criteria [46], and international guidelines [47]. In the majority of instances the diagnosis of cirrhosis was based on historical, clinical, laboratory and radiological variables; histological confirmation was available in a minority from liver biopsy material obtained *via* the percutaneous route (Table 2). The diagnosis of HCC was based on historical, clinical, and laboratory variables together with, as recommended, multimodal imaging (Table 3). The 304 healthy blood donors, who acted as controls, were recruited at the Azienda di Rilievo Nazionale ad Alta Specialiazzione (A.R.N.A.S.) Civico Hospital of Palermo, Italy. Controls were only included if they were born in and continued to reside in Sicily; were aged > 30 years and in line with International guidelines [48, 49] were negative for HBsAg, anti-HCV and anti HIV antibodies and had normal routine blood test results.

All included cases and controls provided written informed consent. This research was approved by the Ethics committee of the Policlinico Hospital (Palermo, Italy).

### DNA extraction and genotyping

Genomic DNA was extracted from whole blood using the WizardGenomic DNA Purification Kit (Promega). DNA quality was assessed using gel electrophoresis (0.8% agarose gel; 5 volts/cm for 1 hour; 1× Tris-borate-EDTA (TBE) buffer; 100 bp and 1kb DNA Ladder (Promega, UK). Genotyping for rs430397 in *GRP78* and rs738409 in *PNPLA3* was performed using the K-Biosciences Competitive Allele Specific PCR (LGC Genomics, Hoddesdon, UK) platform with amplification and detection undertaken using a LightCycler® 480 real-time PCR system (Roche Molecular Diagnostics, Burgess Hill, UK). Genotype calling was performed automatically using proprietary software with minor manual editing of genotype calls. Approximately 12% of samples, randomly selected *a priori*, were genotyped in duplicate to ensure consistent genotype calling. The primers used for KASPar genotyping are detailed in Supplementary Table 1.

### Statistical analysis

#### Genetic analysis

Tests for genetic association, missingness, deviation from Hardy-Weinberg equilibrium and epistasis were performed using PLINK (version 1.9) [50]. Genetic association analyses were performed using logistic regression utilizing additive, dominant and recessive models when comparing HCC cases and controls. Tests for association between demographic covariates were performed under an additive model using the Fisher's exact test to assess significance. Statistical analyses were performed in R [51].

#### Prediction modelling

A two-step analysis comprising of variable selection and decision tree construction was performed using a machine learning technique to establish a rule for predicting phenotypes starting from the genotypes of both the rs430397 and rs738409 variants. In the first step the variables to be used in the second step were selected utilizing a stepwise search, which performs a greedy forward or backward search through the space of HCC case characteristics or attributes. The selection is based on a decision tree classifier for estimating the accuracy of the chosen variable subset and is stopped when the addition/deletion of any remaining attribute resulted in a decrease in evaluation. The second step involved the construction of a tree-like graph or model of decisions based on the previously selected variables. This model exhibits a flowchart-like structure in which each internal node represents a ‘test’ on an attribute, each branch represents the outcome of the test and each leaf node represents a class label. The paths from root to leaves represent the classification rules. The evaluation of the classifier is based on the overall misclassification rate (MCR); the lower the MCR the better the prediction modelling. Both steps were performed using the RWeka package (R-3.2.3 software).

## SUPPLEMENTARY TABLE


