# Supplementary material for: The association of variants in PNPLA3 and GRP78 and the risk of developing hepatocellular carcinoma in an Italian population
Source: Oncotarget. 2016 Nov 24;7(52):86791–802. doi: 10.18632/oncotarget.13558 (PMC5349954; doi:10.18632/oncotarget.13558)
Supplement: Supplementary file 1 [file oncotarget-07-86791-s001.pdf]

The association of variants in *PNPLA3* and *GRP78* and the risk of developing hepatocellular carcinoma in an Italian population

SUPPLEMENTARY TABLE

Supplementary Table S1: Genotyping Primers used in the present study

SNP	Primer	Nucleotide Sequence (5'-3')
rs430397	Allele Specific Forward 1	GAAGGTGACCAAGTTCATGCTAGATAACAGACATCACAGTAACCATG
	Allele Specific Forward 2	AAGGTCGGAGTCAACGGATTCTTAGATAACAGACATCACAGTAACCATA
	Reverse	ATAGTAGACCGGAACAGATCCTAGAAAA
rs738409	Allele Specific Forward 1	GAAGGTGACCAAGTTCATGCTCCTTGGTATGTTCCTGCTTCATC
	Allele Specific Forward 2	GAAGGTCGGAGTCAACGGATTCCTTGGTATGTTCCTGCTTCATG
	Reverse	CGCCTCTGAAGGAAGGAGGGAT
